# Exploring the Utilization of Imaging Modalities in the Diagnosis of Basal Cell Carcinoma: A Scoping Review

**DOI:** 10.7759/cureus.56047

**Published:** 2024-03-12

**Authors:** Mahi Basra, Lucas Shapiro, Hemangi Patel, Collin Payne, Brett Brazen, Alejandro Biglione

**Affiliations:** 1 Osteopathic Medicine, Nova Southeastern University Dr. Kiran C. Patel College of Osteopathic Medicine, Clearwater, USA; 2 Sports Medicine, Nova Southeastern University Dr. Kiran C. Patel College of Osteopathic Medicine, Fort Lauderdale, USA; 3 Dermatology, Broward Health Medical Center, Fort Lauderdale, USA; 4 Internal Medicine, Wellington Regional Medical Center, Wellington, USA

**Keywords:** reflectance confocal microscopy, optical spectroscopy, dermoscopy, bcc, basal cell carcinoma

## Abstract

Basal cell carcinoma (BCC) is a common skin cancer that occurs due to various genetic and environmental factors. Diagnosis is made by a combination of clinical appearance, biopsy, imaging, and histopathological analysis. This review describes the current array of imaging modalities available to physicians to aid in the diagnosis of BCC. It is important to stay up-to-date with improvements in diagnostic screening, and knowledge of these options is instrumental in providing the best care to patients. Embase, Medline Industries, and PubMed were searched for articles within the past 10 years based on a search query that looked for imaging modalities used in the diagnosis and evaluation of a variety of dermatologic conditions. The search was further refined to focus on BCC and satisfy the inclusion/exclusion criteria determined by the authors. The research process was detailed in the Preferred Reporting Items for Systematic Reviews and Meta-Analyses diagram.

Dermoscopy is a non-invasive in vivo microscopic technique used to evaluate skin lesions. Features of dermoscopy cannot be visualized with the naked eye, and studies found that dermoscopy increased diagnostic accuracy. Reflectance confocal microscopy (RCM) examines skin morphology, and recent studies found that 100% of patients with BCC had tumor-free margins when diagnosed with RCM. It allows for a one-stop-shop for diagnosis. Optical spectroscopy samples multiple sites without removing tissue. It helps detect subtle biophysical differences, allowing for earlier diagnosis. High-frequency ultrasound (HFUS) helps determine tumor size, structure, depth of invasion and spread. Studies found statistically significant positive correlations between depth of spread and HFUS readings. Optical coherence tomography takes cross-sectional images to analyze histopathology and morphology. It produces high-resolution images, confers slightly more accurate results than a biopsy, and expedites the treatment process through an earlier diagnosis without a biopsy.These results will advance the fields of dermatology and radiology as they describe unique uses for these imaging modalities. There are a variety of ways to use microscopy, and these techniques may be applied to many different lesions and help revolutionize the diagnosis and treatment of skin cancer and other lesions without the need for multiple, sometimes disfiguring surgical procedures. With the increase in diagnostic accuracy and decrease in diagnosis time, advanced imaging studies will become an integral part of dermatologic diagnosis and be included in future management and treatment plans, especially in the case of BCC.

## Introduction and background

Basal cell carcinoma (BCC) is a type of skin cancer found in humans. It is the most common cancer in the world, with a 20% risk of developing BCC in one’s lifetime in the United States. It occurs due to a combination of phenotypic, environmental, and genetic factors. Risk factors include light hair, skin, and eyes; male sex; an increased number of moles; significant burns and sun exposure; and radiation exposure. There are more than 26 different subtypes of BCC. The four most common subtypes include nodular, superficial, morpheaform, and fibroepithelial BCC, with the nodular subtype being the most common. The likelihood of metastasis and/or death is most strongly predicted by extension beyond fat in lesions 2 cm or greater (28.6 fold increase). This risk of metastasis and death is 50% in patients with nevoid BCC syndrome, whereas unaffected patients with a history of radiation therapy have a 22% chance of metastasis and death. In the case of metastasis, the most common location is lymphatic spread (53%). Typical features on physical examination include a pearly appearance, arborizing vessels, and rolled borders [1]. Aside from clinical diagnosis with the naked eye, various non-invasive imaging techniques may be used in the diagnosis of BCC. The modalities addressed in this review include dermoscopy, reflectance confocal microscopy (RCM), optical spectroscopy (OS), high-frequency ultrasound (HFUS), and optical coherence tomography (OCT). These methods are used to assess primary, recurrent, and residual lesions after treatment. This scoping review aims to identify and describe these imaging techniques to provide the healthcare community with a comprehensive resource regarding the diagnosis of BCC at all stages of the disease.

## Review

Methods

Search Strategy and Selection Criteria

EMBASE, Medline Industries, and PubMed databases were used to perform a literature search on November 1, 2023. The Preferred Reporting Items for Systematic Reviews and Meta-Analyses (PRISMA) statement by the Cochrane Collaboration was used. The following criteria were used to determine which articles to include: human patient population, primary study, clinical trial, cohort study, or cross-sectional trial. All included articles were published between January 1, 2013, to November 1, 2023. Exclusion criteria were articles not written in English, review articles, or animal studies. These criteria were selected to fulfill the objectives of this review.

Key Terms

The key terms used to search for articles were: dermatology, derm, skin lesions, skin imaging, confocal microscopy, laser microscopy, microscopy, OCT, tomography, interferometry, ultrasound, Raman spectroscopy, spectroscopy, fluorescence imaging, dermoscopy, machine-based learning, machine learning, monoclonal B lymphocytosis (MBL), multispectral optoacoustic tomography, multispectral imaging, hyperspectral imaging, and multiphoton tomography. Databases were searched using the Boolean operators “AND” and “OR” as follows: (“dermatology*” OR (“derm*” OR “skin lesions*” OR “skin imaging*”)) AND (“confocal microscopy*” OR “laser microscopy*” OR “microscopy*” OR “OCT*“ OR “tomography*” OR “interferometry*“ OR “ultrasound*” OR “Raman spectroscopy*“ OR “spectroscopy*” OR “fluorescence imaging*“ OR “dermoscopy*“ OR “machine-based learning*” OR “machine learning*“ OR “MBL*“ OR “multispectral optoacoustic tomography*” OR “multispectral imaging*“ OR “hyperspectral imaging*“ OR “multiphoton tomography*“)). These key terms were selected based on current practices utilized in dermatology. Articles were then further screened to include articles discussing BCC.

Evaluation Process

**Figure 1:**
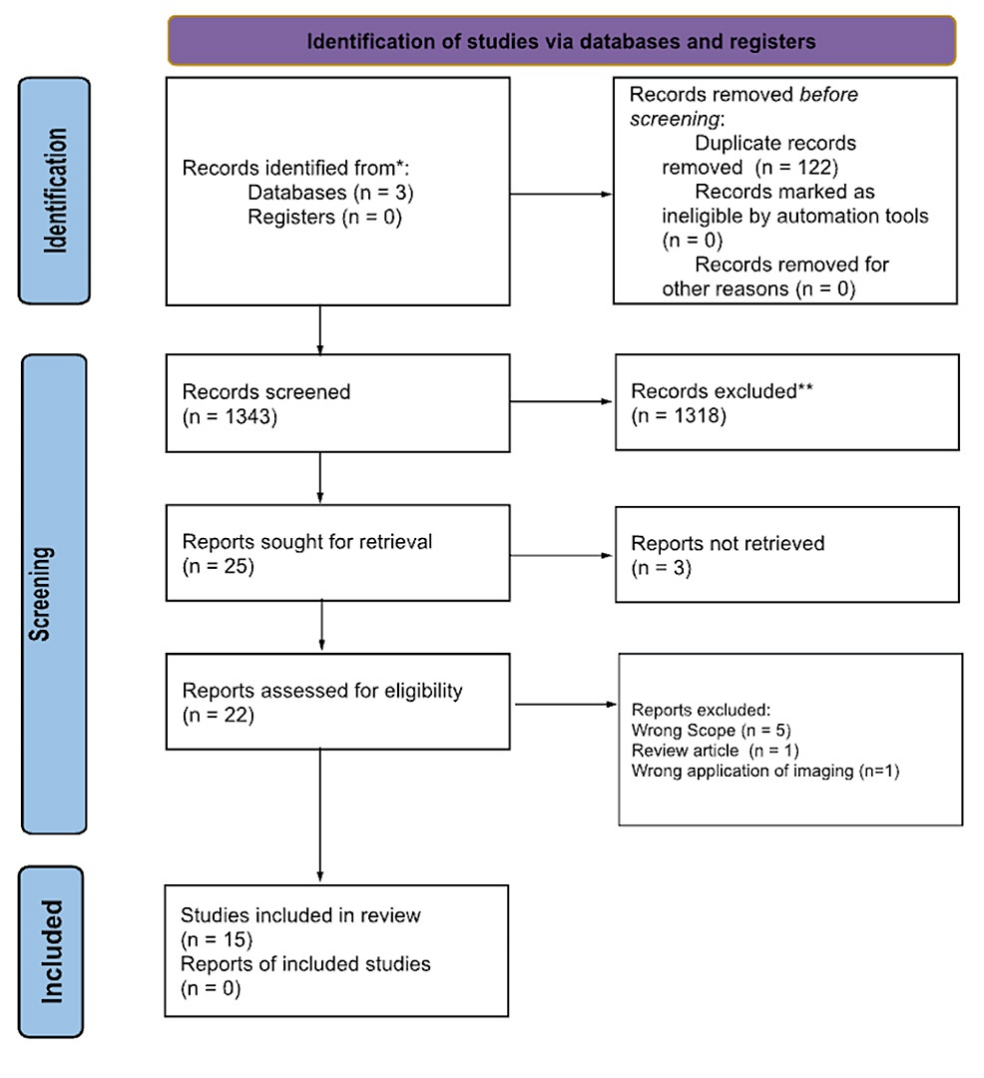
PRISMA diagram PRISMA: Preferred Reporting Items for Systematic Reviews and Meta-Analyses

The process of inclusion for the articles was portrayed in the PRISMA (Figure 1). The authors (LS and MB) evaluated the same 1343 articles after 122 duplicates were removed. Based on inclusion and exclusion criteria, 1318 articles were excluded, and 25 were sought for retrieval. Twenty-two were obtained and assessed for eligibility. The authors (LS, MB, HP, and CP) each independently reviewed the full-text publications. Due to further analysis, five articles were excluded because the article content was considered out of the scope of this review, one article was a review, and one article did not discuss the imaging technique as it relates to the diagnosis of BCC. Disagreements were resolved by discussion with all the aforementioned authors, yielding 15 total studies included in the review. Additional reference lists were not utilized.

Data Collection

Each article was evaluated, and a results table was created with the following columns: author, publication year, study design, sample size, outcomes, study limitations, and future recommendations. The scoping review was written after the data was compiled and each article was analyzed accordingly.

Results

Dermoscopy

Dermoscopy is a non-invasive, in vivo skin surface microscopy technique used to evaluate suspicious skin lesions. A dermatoscope is a magnifying lens with built-in illumination. It allows for transillumination of the lesions to visualize the lesions’ features. BCC features of dermoscopy include arborizing vessels, blue and pink stromal hues, translucency, blue and gray ovoid nests, and leaf-like structures in pigmented lesions [1].

In a prospective observational study, dermoscopy was utilized to analyze whether diagnostic accuracy can be improved when differentiating squamous cell carcinoma (SCC) and BCC in comparison to clinical diagnosis. Out of 154 cases, 141 cases were diagnosed correctly with an accurate clinical impression. Thirteen cases were misdiagnosed. Diagnostic accuracy was improved with dermoscopy. The odds ratio (OR) was overall 2.857 when dermoscopic photos were used compared to clinical photos (p = 0.0049) [2]. Similarly, Wolswijk et al. performed a diagnostic cohort study to compare clinical and dermoscopic evaluation (CDE) to CDE with OCT in BCC detection. Out of 100 patients, sensitivity was 100% for CDE-OCT and 60% for CDE (p = 0.005). CDE-OCT had a specificity of 95%, while CDE was 96.3% (p = 0.317) [3].

Reflectance Confocal Microscopy

RCM, or confocal imaging, is a non-invasive imaging tool that can be used to diagnose skin lesions. A confocal microscope is used to capture images non-invasively. Nuclear and cellular skin morphology is analyzed with both lateral and axial resolution [4].

Kadouch et al. conducted a prospective cohort study with 100 patients who had clinically suspected BCC. Two study groups were formed: (1) diagnosis and surgical excision with RCM; and (2) punch biopsy diagnosis and planned excision. RCM and punch biopsy sensitivities were similar, at 100% and 93.9%, respectively. Punch biopsy had increased specificity to 79% when compared to RCM's 38%. No adverse events were noted in either group. Thus, RCM and punch biopsy have similar accuracy in diagnosing BCC [5]. Similarly, Woliner-van der Weg et al. determined that RCM and punch biopsy had an equivocal sensitivity of 99% when diagnosing 288 patient lesions. RCM had 59.1% specificity, while punch biopsy had 100%. Patient satisfaction was comparable in both methods [4]. Biopsy outperforms RCM in terms of specificity [1,4]. RCM may also help improve patient satisfaction by providing a “one-stop shop” to improve tumor-free margins. One hundred percent of 40 patients diagnosed with RCM had tumor-free margins when compared to 94% of BCC patients treated with a punch biopsy and a consecutive planned excision [6].

Optical Spectroscopy

OS is a non-invasive diagnostic method that can be used as a screening tool for suspicious skin lesions. OS allows multiple-site sampling without tissue removal, immediate feedback, and identification of biophysical features. The Zenalux OS device (Zenalux Biomedical, Inc., NC, USA) has been utilized in the past to distinguish breast malignancy and healthy tissue but has not been extensively used in skin biopsies [7].

Carpenter et al. performed a study on 18 patients, analyzing 27 lesions to assess the efficacy of OS in the examination of potentially concerning skin lesions and determining biopsy necessity. This study concluded that OS had a 44% correct prediction in differentiating benign versus malignant lesions. Clinical suspicion demonstrated 54% sensitivity and 92% specificity. There was an absolute difference of 29% between benign and malignant lesions. OS allows the examination of multiple skin lesions without a biopsy, offering real-time feedback. Detection of subtle biophysical features allows earlier detection before these characteristics become apparent on macroscopic examination. Additionally, OS findings suggested that both BCC and SCC are hypoxic when compared to normal skin [7].

High-Frequency Ultrasound

HFUS is a non-invasive technology that facilitates the assessment of tumor size and structure, measurement of depth of spread, and BCC location in relation to surrounding tissue. This data allows localization of BCC in areas where the risk of recurrence is high to allow for surgical treatment and other non-invasive modalities. Typically, transducers with a 20-100 MHz frequency are used; however, it has been reported that high resolutions (20 MHz and higher) have been used to determine the depth and boundaries of BCC. Other studies have also reported diagnostic utility in transducers utilizing medium frequency (10-14 MHz) [8].

A comparative study was conducted by Khlebnikova et al. to evaluate HFUS imaging with 30 MHz and 75 MHz probes to compare the depth spread of BCC. Nineteen patients with a total of 27 pathological lesions were examined utilizing HFUS with 75 and 30 MHz probes with a resolution of 21 and 48 μ and scan depths of 4 to 8 mm, respectively. The tumor depth spread was measured and biopsy material was taken either through a punch biopsy or surgical excision. After morphological examination, the tumors were divided into two categories: thin (invasion depth less than or equal to 1 mm) and thick (invasion depth greater than 1 mm). Results concluded that thin BCC had a statistically significant correlation between histological results and 75 MHz HFUS measurements (r = 0.870). A high, statistically significant (r = 0.951) correlation was found with thick BCC depth measured with the 30 MHz transducer [8].

Optical Coherence Tomography

OCT is a non-invasive diagnostic method for BCC and other dermatologic lesions. OCT generates real-time cross-sectional images with 1.5 mm depth that display the histopathological and morphologic features of the lesions [2]. OCT utilizes light interferometry to identify morphological characteristics. Advantages of OCT include higher image resolution, increased imaging depth, and rapid diagnosis [3,9]. While a biopsy can also be used, OCT does not cause scarring, infection, or rebleeding [2-3]. Punch biopsy is generally used to guide the diagnosis and treatment of BCC lesions. A prospective study conducted by Adan et al. assessed whether OCT can replace punch biopsy in BCC diagnosis. Five hundred fifty-three patients over the age of 18 were enrolled in either an OCT group or a punch biopsy group. According to histopathology, 75% of 299 lesions in the OCT group had a correct diagnosis when compared to 72% in the punch biopsy group. Within the OCT group, a confident diagnosis of BCC or its subtype was achieved in 66% of patients. The remaining 34% of individuals required an extra biopsy for a conclusive diagnosis. Among the 225 histologically confirmed BCC lesions in the OCT group, 192 were successfully identified with high confidence, resulting in a sensitivity of 85% and a specificity of 94%. Among the 192 BCCs identified by OCT, OCT correctly identified 47 of the 72 superficial BCCs (65% specificity). No significant difference was noted in treatment cost, post-treatment cost, or total mean cost between both the OCT and punch biopsy groups. Thus, Adan et al. concluded that OCT is an economical approach with slightly higher efficacy (0.94 vs. 0.93) when compared to punch biopsy, respectively, without compromising the standard of care or patient safety [10].

Clinical and dermatoscopic examination (CDE) is used in conjunction with OCT, which may improve detection rates of BCC lesions that recur. Wolswijk et al. conducted a diagnostic cohort study to compare the accuracy of CDE and the combination of CDE and OCT in detecting recurrent or residual BCC lesions after topical treatment of superficial lesions. In 100 patients, CDE-OCT had 100% sensitivity and 95% specificity (p = 0.005), while CDE had 60% sensitivity and 96.3% specificity. The negative predictive value for CDE was 90.6% (77 out of 85 patients) and 100% (76 out of 76 patients) for CDE-OCT. When compared to CDE alone, CDE-OCT yielded significantly higher accuracy and the ability to detect recurrent BCC lesions after topical treatment [2]. Adjunctively, line-field confocal (LC)-OCT can aid in improving diagnostic accuracy in clinically unsure cases of BCC. LC-OCT was used to analyze 182 lesions in 154 patients. LC-OCT had 98% sensitivity and 80% specificity when compared to histologic analysis. The combination of dermoscopy and LC-OCT had 100% sensitivity and 81.2% specificity. Gust et al. concluded that LC-OCT allows for improved diagnostic confidence, sensitivity, and specificity. It is useful in diagnosing clinically equivocal lesions, which may be difficult to diagnose with only dermoscopy. LC-OCT can be influenced by image quality but not by anatomic location [11]. Similarly, a prospective study concluded that the combination of OCT and clinical examination of patients slightly improved their ability to discriminate between non-superficial and superficial BCC types in the analysis of 250 lesions (sensitivity of 97.6% and 95.2%, respectively) [12]. OCT adjunct use increased the detection of recurrent BCC when compared to clinical examination with dermoscopy by 12.2% [11]. Markowitz et al. conducted a similar study and concluded OCT increases diagnostic accuracy relative to clinical and dermoscopic examination (57% accuracy in clinical examination; 70% accuracy for dermoscopy; and 88% accuracy in OCT). OCT can rule- out BCC, thus necessitating rapid surgical intervention without having to wait for biopsy results [13].

High-definition (HD) OCT offers a real-time horizontal image in addition to the traditional vertical OCT. Allowing for immediate visualization of both dimensions of lesions, HD-OCT parallels the “Munich method” of cutting frozen tissue in horizontal sections in micrographic surgery. Maier et al. evaluated 80 HD-OCT images of 20 BCC samples. In 45% of samples, HD-OCT corresponded exactly to the histological results obtained for comparison. HD-OCT had a sensitivity of 74% and a specificity of 64% [14]. OCT as an adjunctive supplementary diagnostic tool can help to treat BCCs early and decrease patient mortality [15]. The results of the studies included are summarized in Table 1.

**Table 1:** Results table OCT: optical coherence tomography; BCC: basal cell carcinoma; SCC: squamous cell carcinoma; LC-OCT: line-field confocal optical coherence tomography; RCM: reflection confocal microscopy; CE: clinical examination; HFUS: high-frequency ultrasound; HD-OCT: high-definition optical coherence tomography; CDE: clinical and dermatoscopic examination; OS: optical spectroscopy

Reference	Study Type	Sample Size	Aim	Findings	Limitations	Recommendations
Carpenter et al., 2018 [7]	Prospective pilot study	18 patients, 27 lesions	Evaluate the utility of OS in the screening of non-melanoma suspicious skin lesions and determine biopsy need	OS displayed utility in observing multiple lesions without biopsy, provided immediate feedback, and could detect subtle biophysical features before they appeared on a gross exam. Findings suggest BCC and SCC display a relative decrease in total hemoglobin concentration compared to internal control (i.e., normal skin)	Non-representative patient population. Limited clinical availability, cost, device complexity, and training. Only two physicians were involved in the pre-biopsy examination	Repeat study design in a real-world primary analysis of suspicious lesions in the setting of a diverse and representative population
Gust et al., 2022 [11]	Prospective study	154 patients, 182 lesions	Investigate the accuracy of LC-OCT in diagnosing equivocal cases of BCC compared to dermoscopy alone	The diagnostic accuracy of LC-OCT in ruling out BCC was 91%, compared to 88% with dermoscopy alone. The diagnostic accuracy of LC-OCT for all BCC subtypes was 90%. The combination of dermoscopy and LC-OCT yields a diagnostic accuracy of 92.9% with all lesions; the accuracy increases to 98.2% in confident cases	A reduced number of non-BCC lesions could distort overall specificity	Consider repeating the study with LC-OCT utilization as the primary analysis of suspected BCC lesions within defined anatomical borders to define specific characteristics of BCC by body region
Heath and Bar, 2023 [1]	Review	Variable	Discuss the epidemiology, staging, and modalities for diagnosis and treatment (excision and medication)	OCT exhibited a sensitivity ranging from 79% to 94% and specificity ranging from 85% to 96% per several studies. Summated meta-analyses reveal dermoscopy has a sensitivity of 89-91% and a specificity of 95%. Experienced RCM operators obtained a sensitivity of 91.7% and a specificity of 91.3%	Not applicable	Not applicable
Hussain et al., 2016 [15]	Prospective cohort	58 patients	Assess the utility of adjunct OCT in monitoring for BCC recurrence, compared to dermoscopy and clinical examination alone	Adjunct OCT was able to detect six recurrent BCCs that dermoscopy and CE alone did not detect	Small sample size. The absolute diagnostic accuracy of OCT was not assessed	Consider utilizing OCT for BCC recurrence surveillance over a prolonged period of time with a more robust sample size
Kadouch et al., 2017 [5]	Prospective cohort study	100 patients	Assess the diagnostic utility of RCM in the diagnosis and subtyping of clinically suspicious BCC	RCM displayed a higher sensitivity compared to biopsy (100% vs. 93.94%), but the biopsy was more specific (100% vs. 75%). Agreement between RCM and excision for subtyping was between 50-85% and 77% for biopsy	RCM utility varies greatly depending on user experience, with a specificity of 38% in those with <1 year of experience, increasing to 75% with 10+ years of experience	Repeat the study with an emphasis on determining specific criteria to improve BCC subtype differentiation
Kadouch et al., 2017 [6]	Randomized controlled trial	95 patients	To assess the efficacy of RCM in a “one-stop-shop” environment compared to that of standard treatment in the management of clinically suspected BCC	Utilizing a "one-stop-shop" with RCM capabilities improved tumor-free margins: 100% (40/40) tumor-free margins in the one-stop-shop arm versus 94% (31/33) tumor-free margins in the standard treatment arm. The mean time for RCM was 13 minutes, compared to a maximum of a two-week wait period for PB results in the standard treatment group	Study not conducted in the USA, discouraging immediate implementation	Repeat the study with a more robust sample size and consider evaluating the efficacy of RCM versus standard care when both treatment arms are one-stop-shop settings
Khlebnikova et al., 2020 [8]	Comparative study	27 BCC	Assess the depth of invasion of histologically verified BCCs with HFUS using 30-MHz and 75-MHz probes	After lesions were resected and histologically analyzed, a positive correlation coefficient was found between thin (≤1 mm) invasion depth BCCs and the 30-MHz probe (r = 0.870). A positive correlation coefficient was also found between the thick (>1 mm) invasion depth BCCs and the 75-MHz probe (r = 0.951)	Small sample size. Intra- and inter-examiner reliability was not assessed	Repeat the study with a more robust sample size and evaluate the HFUS utility for guiding Mohs micrographic surgery
Maier et al., 2014 [14]	Retrospective observational, pilot study	20 BCC biopsies	Evaluate the utility of HD-OCT in analyzing ex vivo BCC biopsies compared to histological analysis	In 45% of evaluated tumors, HD-OCT correctly analyzed all four sides of the sample. In 10% of evaluated tumors, HD-OCT did not correctly analyze any of the sample’s sides. Overall, HD-OCT specificity was 64%, and sensitivity was 74%	Very small sample size. Secondary analysis	Repeat secondary analysis with a more robust sample size to evaluate the feasibility of an en vivo analysis
Markowitz et al., 2015 [13]	Observational	115 lesions	Assess the diagnostic accuracy of OCT for BCC	Utilization of OCT resulted in a statistically increased sensitivity (93%) and specificity (80%) compared to dermoscopy. The authors’ estimated reduction in biopsy necessity is a 36% reduction	Inherent limitations of observational studies and sole inclusion of “clinically difficult” lesions	Increase the sample size and consider utilizing a prospective study design with all non-pigmented lesions included
Olsen et al., 2016 [9]	Retrospective cohort	142 images	Evaluate the diagnostic utility of OCT in both BCC and actinic keratosis, and assess their ability to differentiate from each other and normal skin	Experienced OCT operators (≥ 1 year) were able to detect BCC with a sensitivity of 86-95% and a specificity of 81-98%	The study was performed with single-frame images without patient communication/clinical assessment	Consider repeating the study with multislice imaging techniques to assess the ceiling of diagnostic accuracy
Ryu et al., 2018 [2]	Prospective observational	154 cases	Characterize the factors that cause clinical misdiagnosis between BCC and SCC; and evaluate whether dermoscopy is able to improve correct diagnosis rates	Of the 154 cases evaluated, 13 cases were inversely diagnosed; the authors found the following contributing factors: “scales without both pigmentation and rolled border were favored for SCC, but BCC was favored vice versa. Ulceration, telangiectasia, and translucency contributed as confusing factors for proper diagnosis.” Dermoscopy allowed an improved diagnostic accuracy of OR 2.86	Smaller sample size of discrepancies. Three out of six expert reviewers were residents and not board-certified	Repeat the study to include a larger sample size with more board-certified expert reviewers
Sinx et al., 2020 [12]	Prospective cohort study	182 patients, 250 lesions	Evaluate the diagnostic utility of OCT in characterizing primary BCCs compared to clinical examination	With the utilization of OCT alongside CE, the specificity of BCC diagnosis increased to 76.8%, with a similar sensitivity to CE alone. Additionally, OCT alongside CE showed an improved ability to discriminate between superficial and non-superficial BCCs	The first 150 OCT scans were omitted for training purposes in order to learn technology, highlighting the significant learning curve of OCT operators	Assess the specificity of OCT alongside CE with a much larger cohort and with training scans included to evaluate its true utility and implementation capabilities
Woliner-van der Weg et al., 2021 [4]	Randomized controlled trial	288 patients	Determine the diagnostic accuracy of RCM versus punch biopsy in the primary analysis and subtyping of BCC. Additionally, to evaluate patient preferences between the two modalities	Overall, the sensitivity between RCM and PB was similar in the diagnosis of BCC. RCM displayed poorer sensitivity in the aggressive subtype of BCC but had a similar sensitivity compared to RCM in the diagnosis of the non-superficial subtype. Patient satisfaction was comparable between the two groups	Cost and experience required for RCM. Additionally, all study participants were at hospitals in the Netherlands	Conduct additional studies to determine if RCM is a practical alternative for specific subtypes of BCC
Wolswijk et al., 2023 [3]	Diagnostic cohort study	100 patients	Assess the diagnostic accuracy of CDE compared to CDE with OCT in the detection of recurrent or residual BCC after topical treatment of the superficial BCC	CDE with OCT displayed a sensitivity of 100% in the detection of recurrent/residual BCC compared to 60% with CDE alone (p = 0.005). Additionally, the negative predictive value of CDE with OCT was 100%, while CDE alone was 90.6% (p = 0.005)	A small number of OCT operators and histopathological verification only occurred in nine patients in the CDE-OCT group	Repeat the study with a complete histopathological analysis of all patients to improve validity
Adan et al., 2022 [10]	Multi-center randomized controlled trial	533 patients	Assess whether OCT can reduce the need for punch biopsy in the management of BCC	In the OCT group, there were 225 histologically verified BCCs (or BCC subtypes). Of these verified lesions, 192 were detected, with a high confidence level of BCC diagnosis correlating with a sensitivity of 85% and a specificity of 94%. 94.6% of non-BCC lesions were detected as non-BCC lesions without a significant difference in total mean costs. These findings support the idea that OCT is non-inferior to a punch biopsy in the diagnosis of BCC	An OCT diagnosis was made by a single, experienced, OCT-trained physician. Patients with large lesions or lesions located in high-risk zones of the face were excluded due to the contour-related challenges of the OCT operation	Repeat the trial with a real-life representative group of OCT operators and consider analyzing high-risk zones of the face/neck to define efficacy in these regions

Discussion

BCC is a skin cancer that is the most common cancer found in humans [1]. There are risk factors, including but not limited to light hair, skin, and eyes; male gender; the presence of multiple moles; burns; sun exposure; and radiation exposure [1]. The common features on a physical exam include a pearly appearance, rolled borders, and arborizing vessels [1]. Aside from the naked eye of a clinician, there are many other diagnostic modalities, such as dermoscopy, RCM, OS, HFUS, and OCT.

Imaging Modalities

Dermoscopy is a non-invasive, in vivo skin surface microscopy technique that aids in screening skin lesions in great detail [1]. The common features of BCC include arborizing vessels, blue and pink stromal hues, translucent, blue, and gray ovoid nests, and leaf-like structures in pigmented skin lesions, all of which can be evaluated with dermoscopy [1]. Although some of these features are diagnosed with the naked eye, a prospective observational study comparing clinical diagnosis to dermoscopy use showed 13 misdiagnoses with the naked eye [1].

RCM is a non-invasive imaging tool for skin lesions. It captures images and analyzes the nuclear and cellular skin morphology in lateral and axial resolution patterns [4]. When surgical excision with RCM was compared to punch biopsy with planned excision, similar accuracy and sensitivity in diagnosing were prevalent, and patient satisfaction was equivalent [4,5]. In the study conducted focusing on tumor-free margins, punch biopsy had 94% of patients with tumor-free margins, while RCM had 100% of patients with tumor-free margins. Overall, punch biopsy shows greater promise in comparison to RCM for accurately diagnosing skin lesions.

Additionally, OS is a screening tool that examines multiple sites without tissue removal and provides great detail on biophysical features [7]. The ability to detect biophysical features without the removal of tissue provides comfort to patients and facilitates early detection, preventing its progression.

HFUS is a non-invasive technology that assesses the tumor size and structure and measures the depth of spread and location in comparison to the surrounding tissue [8]. HFUS determines where the risk of recurrence is high, so treatment can be specific to the region of concern. Tumors are thin (depth less than or equal to 1 mm) or thick (depth greater than 1 mm). The thin BCC tumors evaluated with HFUS showed a positive correlation with histology at 75 MHz HFUS measurements. The thick BCC tumors showed a positive correlation in results with histology at 30 MHz HFUS measurements. The high statistically significant correlations display the use of HFUS when histology is unavailable or inaccessible, as it is a very time-consuming process for diagnosis.

OCT is a diagnostic method that creates cross-sectional images with a 1.5 mm depth of the histopathological and morphological features of the lesions [3]. When compared to a punch biopsy, OCT is less invasive and prevents the possibility of scarring, infection, and bleeding as seen in biopsies [3]. This reduces the likelihood of patient dissatisfaction. In a study evaluating OCT and punch biopsy, 75% of lesions in the OCT group had a correct diagnosis, while 72% in the punch biopsy had a correct diagnosis [5]. The OCT group was found to have a 66% rate of diagnosis accuracy, 85% sensitivity, and 94% specificity [5].

When OCT is used alongside CDE, the rate of BCC lesion detection is improved, and the ability to detect superficial versus non-superficial BCC types is greater [5]. The study conducted by Wolswijk et al. evaluated the accuracy of CDE alone and CDE with OCT, resulting in a 60% sensitivity and 96.3% specificity in CDE, while CDE-OCT had a 100% sensitivity and 95% specificity. CDE-OCT was found to be more accurate when compared to CDE alone. LC-OCT had a sensitivity of 98% and a specificity of 80% when compared to histologic analysis, and when LC-OCT was combined with dermoscopy, there was a 100% sensitivity and 81.2% specificity [4]. Thus, LC-OCT has greater sensitivity and specificity and is useful in diagnosing equivocal lesions, which are generally difficult with dermoscopy alone [4]. Another subtype of OCT is HD-OCT, which offers horizontal images in addition to the traditional vertical OCT, improving the detection of BCC [12]. OCT can rule out BCC, which can prevent the need for biopsy and accelerate surgical intervention, improving patient prognosis.

Limitations

When evaluating medical literature, it is critical to consider the limitations of the studies and how this impacts the interpretation of the results. In this review, some imaging modalities may have been missed because only three databases were surveyed, and articles in other languages than English were excluded. Another common limitation of the included articles is the small sample size. It is important to be careful when applying the results of smaller studies to the general population. Additionally, some earlier studies may have been missed due to the 2013 cutoff, but this decision was made to include the most recent and up-to-date data in this field. Some terms that could have been included to pinpoint more articles specific to BCC include "nodular, superficial, morpheaform, and fibroepithelial BCC.”

In terms of OS, there is limited clinical availability as the device is costly and requires advanced training for proper use [7]. Creating a list of criteria for physicians to look out for would be a helpful way to streamline the diagnosis of BCC using this modality.

## Conclusions

BCC is the most common cancer in the world and has several subtypes. Aside from clinical diagnosis, other imaging techniques may be utilized in BCC diagnosis. This review aims to analyze and describe various imaging modalities used to diagnose BCC. BCC diagnosis can be done through invasive methods such as the gold standard, punch biopsy, or other non-invasive imaging that may reduce adverse effects on patients. Imaging methods include dermoscopy, RCM, OS, HFUS, and OCT. In this review, studies detailing the efficacy of these modalities are summarized to provide a cohesive manuscript that physicians can use to explore different options when diagnosing BCC. Utilization of these non-invasive imaging techniques may improve diagnostic accuracy, decrease unnecessary procedures, minimize the risk of scarring, and guide treatment plans. Additional research is warranted in BCC diagnosis using these techniques to guide healthcare professionals in dermatology in the future. A larger sample size of diverse patients would aid in increasing the accuracy and reliability of these studies. Dermoscopy, RCM, OS, HFUS, and OCT may also be applied to other dermatologic conditions, including other cutaneous cancers. Therefore, physicians can gain more use from these modalities beyond the diagnosis of BCC.
